# Effects of Resistance Training in Hypobaric vs. Normobaric Hypoxia on Circulating Ions and Hormones

**DOI:** 10.3390/ijerph19063436

**Published:** 2022-03-14

**Authors:** Rafael Timon, Guillermo Olcina, Paulino Padial, Juan Bonitch-Góngora, Ismael Martínez-Guardado, Cristina Benavente, Blanca de la Fuente, Belen Feriche

**Affiliations:** 1 Faculty of Sport Sciences, Universidad de Extremadura, 10003 Caceres, Spain; golcina@unex.es; 2 Department of Physical Education and Sport, Faculty of Sport Sciences, Universidad de Granada, 18011 Granada, Spain; ppadial@ugr.es (P.P.); juanbonitch@ugr.es (J.B.-G.); cbenavente@ugr.es (C.B.); mbelen@ugr.es (B.F.); 3 BRABE Group, Faculty of Life and Nature Sciences, Universidad de Nebrija, 28248 Madrid, Spain; imartinezgu@nebrija.es; 4 High Performance Center of Sierra Nevada, Spanish Sport Council, 18196 Granada, Spain; blanca.delafuente@csd.gob.es

**Keywords:** growth hormone, cortisol, inorganic phosphate, bicarbonate anion, resistance training, hypoxia

## Abstract

Hypobaric hypoxia (HH) seems to lead to different responses compared to normobaric hypoxia (NH) during physical conditioning. The aim of the study was to analyze the hormonal and circulating ion responses after performing high-intensity resistance training with different inter-set rest under HH and NH condition. Sixteen male volunteers were randomly divided into two training groups. Each group completed two counterbalanced resistance training sessions (three sets × ten repetitions, remaining two repetitions in reserve), with both one- and two-minute inter-set rest, under HH and NH. Blood samples were obtained to determine hormones and circulating ions (Ca^2+^, Pi, and HCO_3_^−^) at baseline and after training sessions (5, 10, and 30 min). Resistance training with one-minute rest caused greater hormonal stress than with two-minute rest in cortisol and growth hormone, although the hypoxic environmental condition did not cause any significant alterations in these hormones. The short inter-set rest also caused greater alterations in HCO_3_^−^ and Pi than the longer rest. Additionally, higher levels of Ca^2+^ and Pi, and lower levels of HCO_3_^−^, were observed after training in HH compared to NH. Metabolic and physiological responses after resistance training are mediated by inter-set rest intervals and hypoxic environmental condition. According to the alterations observed in the circulating ions, HH could cause greater muscular fatigue and metabolic stress than NH.

## 1. Introduction

Various studies on resistance training under hypoxic conditions have been performed, both to assess acute physiological responses [1,2,3] and long-term adaptations [4,5]. Previous studies have shown that resistance training under hypoxic conditions enhances the build-up of metabolites [6], which clearly have an important role in muscle growth. Resistance training performed under hypoxia lead to a significant oxidative and hormonal stress [6,7]. Increases in anabolic hormones [8,9] and reactive oxygen species [10], as well as alterations in circulating ions (Pi, Na^+^, and H^+^) [3,11], have been observed after the resistance training.

However, there is no unanimity in the results obtained regarding the physiological responses caused since factors such as the type (hypobaric hypoxia [HH] or normobaric hypoxia [NH]) and the severity of the hypoxia (the partial pressure of oxygen [pO_2_] during HH, or the fraction of inspired oxygen [% FiO_2_] during the NH), or the training load (intensity, volume, and recovery), could influence the changes to a greater or lesser extent. Recently, Feriche et al. [3] observed significant increases in growth hormone (GH) and Ca^2+^ and reductions in Pi after resistance training (3 sets × 10 RM, 2 min rest) performed at a moderate terrestrial elevation (2320 m above sea level [m asl]), with lower values after hypoxia compared to normoxia. Likewise, a previous study concluded that low-intensity resistance exercise performed in hypoxia (FiO_2_: 0.15) did not induce greater anabolic hormonal responses than when performed in normoxia [12]. On the contrary, Kon et al. [8] showed that low-intensity resistance training (5 sets × 14 repetitions at 50% of 1RM, 1 min rest) performed in NH (FiO_2_: 0.13) caused greater metabolic and hormonal responses than when performed in normoxia.

On the one hand, terrestrial altitude induces different physiological responses compared to simulated altitude, suggesting that HH is a more severe hypoxic environment [13,14]. Several studies have observed a greater decrease in minute ventilation, alveolar ventilation, and oxygen saturation [13,15], as well as greater oxidative systemic stress [14] with HH compared to NH exposure. However, to the best of our knowledge, there is no research comparing the acute hormonal and metabolic responses in HH with NH after performing resistance training. However, the differences in barometric pressure between HH and NH could affect the metabolic response, as well as the neuromuscular activation and fiber recruitment during resistance training, which could decrease the effectiveness to obtain muscle hypertrophy [7]. The reduced barometric pressure in HH could lead to increased dead space ventilation, resulting in decreased minute ventilation and reduced blood oxygen saturation (SpO_2_), as well as an increased metabolic acidosis [15,16]. Furthermore, the high-threshold motor units could be recruited due to the increased anaerobic metabolism in hypoxic conditions [17,18]. On the other hand, high-intensity multiple sets of resistance training also lead to important acute physiological responses [17,19,20], mediated by the inter-set rest intervals [20,21]. Shorter inter-set rest intervals (60–90 s) seem to cause greater hormonal stress and muscle activation than longer rest periods, both under normoxic [22] and hypoxic [21] conditions. Longer rest periods (180 s) allow clearance of intramuscular metabolites from the circulation, limiting the beneficial stimulus of metabolic stress on muscle activation [21].

Therefore, the aim of the study was to analyze the effect of the hypoxic environment on hormonal and circulating ion responses after performing high-intensity resistance training with different inter-set rest under HH and NH. We hypothesized that high-intensity resistance training with short inter-set rest under HH conditions produces a greater hormonal and metabolic responses than under NH, since the reliance on anaerobic metabolism is increased.

## 2. Material and Methods

### 2.1. Participants

A total of 19 volunteers were recruited for the study, who were randomly divided into two groups. Group 1: *n* = 9 (age: 23.6 ± 3.2 years; height: 177.2 ± 5.7; weight: 73.9 ± 5.3 cm; fat mass %: 10.5 ± 3.8* kg) and Group 2: n = 7 (age: 26.0 ± 3.0 years; height: 174.0 ± 5.0 cm; weight: 73.9 ± 7.8 kg; fat mass %: 15.8 ± 5.0*, *p* = 0.029, compared with fat mass% of G1). There were 3 dropouts (1 muscle injury, 1 acute illness, and 1 suffering from claustrophobia in NH) and finally only 16 participants were evaluated. They had no health or muscular disorders, had not ingested substances to increase muscle size for the previous year, and had not been exposed for more than 3–4 consecutive days to elevations above 1500 m asl at least 2 months before the study. All participants were recreational weightlifters, who performed their own resistance training during the previous 12 months with at least a minimum of 3 sessions per week. Prior to the study, all participants were provided with information detailing the purpose and requirements of the research and provided signed informed consent. This study was approved by the Local University Research Ethics Committee (code 10/18) and conducted in accordance with the Helsinki Declaration, except for registration in a database.

### 2.2. Resistance Training

One week before training, the participants attended the laboratory for baseline anthropometric (height [Seca 202, Seca Ltd., Hamburg, Germany] and body mass [Tanita BC 418 segmental, Tokyo, Japan]) measurements. Afterwards, each participant was tested to determine the 12-repetition maximum (12-RM) for every exercise that would later be performed during training sessions.

During intervention, each group completed two counterbalanced resistance training sessions with 72 h rest between them. Group 1 trained under HH condition at 2320 m asl, with 1-min inter-set rest (HH1) and with 2-min inter-set rest (HH2). HH training was performed in the gym of the Altitude High Performance Center of Sierra Nevada (Spain). Group 2 trained in a training room at 690 m asl under NH condition, with 1-min inter-set rest (NH1) and with 2-min inter-set rest (NH2). Participants under NH condition were connected to a facial mask coupled with an air reservoir bag of 50 L that provided hypoxic air with FiO_2_ = 0.169 (simulated 2320 m asl). The FiO_2_ = 0.169 was produced by a hypoxic generator with a semi-permeable filtration membrane (nitrogen filter technique; CAT 12, Louisville, CO, USA) and it was controlled using an electronic device (HANDI+, Maxtec, Salt Lake City, UT, USA). The simulated altitude was calculated according to the chart and guidelines provided by the hypoxic generator manufacturer.

The training sessions consisted of a warm-up of 15 min of activation, joint mobility, and stretching. After that, a full-body routine with six exercises (barbell back squat, machine leg press, seated cable row, wide grip lat pulldown, bench press, and barbell military press) was performed with a volume of 3 sets × 10 repetitions (remaining 2 repetitions in reserve) per exercise. Cadence of repetitions was carried out in a controlled manner, with a concentric action of approximately 1 s and an eccentric action of approximately 2 s. The load was adjusted for each exercise as needed on successive sets to ensure that subjects achieved the target repetition range (8–10 repetitions). All routines were directly supervised by the research team to ensure proper performance of the respective routines.

### 2.3. Measurements

Before the intervention under fasting condition and after each training session, venous blood samples were taken for determination of Ca^2+^, HCO_3_^−^, Pi, GH, and Cortisol (C). The basal condition for both groups was established from a blood extraction collected 2 days prior to the first training session after 48 h of abstention from training. All preliminary laboratory assessments and basal blood testing were performed in normoxia. Immediately following the training session, the antecubital vein of the arm of each participant was canalized via a catheter, which remained permeable by using physiological saline. A total of 5 mL of blood was extracted at 5, 10, and 30 min after exercise. An amount of 2 mL of blood before each extraction was discarded to avoid dilution of the sample. To minimize bicarbonate loss, specimens were kept tightly capped. In all cases, blood samples were kept in ice (4 °C) and centrifuged in the following 4 h during 10 min at 1790 g (relative centrifugal force). Finally, 500-µL aliquots were stored at −70 °C until processing. Ion determinations were performed in a COBAS C-311 System (Roche, Basel, Switzerland), and hormones were determined in a COBAS E-411 System (Roche, Basel, Switzerland), which was calibrated daily. The COBAS system includes calibration systems for each batch of reagents, and these are validated by comparison with predefined limits for each analyte. Kits that were tested in COBAS had previously been validated by the commercial company and offered the coefficient of variation (CV) of the protocol (CV < 3%).

Blood lactate concentration from the antecubital vein was analyzed by using Lactate Pro 2 (Arkray, Japan) at 3-min post-exercise. The maximum value was registered in mmol/L.

SpO_2_ was measured in duplicate using a pulse oximeter (Wristox 3100; Nonin, Plymouth, MN, USA) at the end of each training session, and the average value was recorded.

Rating of perceived exertion (RPE) was obtained by showing a graphical scale to participants 30 min after completing the training session [23], with a Category Ratio-10 scale.

### 2.4. Statistical Analysis

Statistical analyses were performed using the Statistical Package for Social Sciences (SPSS, version 27.0, Chicago, IL, USA). The Shapiro–Wilks test was applied to verify a normal distribution of data, and Levene’s test was used to assess the homogeneity of variance. A multifactorial ANOVA of repeated measures (within-group factor with four levels: baseline, 5, 10, and 30 min) was independently performed, analyzing the main effects and interaction effects of inter-set rest factor (1-min vs. 2-min) and hypoxic environmental condition factor (HH vs NH) on the variables. Post-hoc Bonferroni tests were performed when appropriate for multiple comparisons. The effect size (ES) was calculated for all dependent variables, taking into account the dispersion of the group means during the different time points. Additionally, a one-way ANOVA was used to compare participant characteristics and control variables. The significance level was set at *p* ≤ 0.05, with a confidence level of 95%. Means and standard deviations (SD) were used as descriptive statistics.

## 3. Results

### 3.1. Control Variables

Table 1 shows the control variables (SpO_2_, lactate, and RPE) that have been measured after training sessions. % SpO_2_ values were significantly lower in HH than in NH, but without significant within-group differences between one- and two-minute inter-set rest. Lactate values (mmol/L) after training were significantly higher in all conditions when comparing HH with NH. There were no significant differences between-groups (HH vs. NH) in RPE, although significantly higher values were observed when a one-minute inter-set rest was used.

### 3.2. Hormones and Circulating Ions

Table 2 shows the circulating ions and hormones at baseline and after training under HH and NH condition. Cortisol increased after training sessions with one-minute rest, both in HH (*p* < 0.015, ES: 0.428) and in NH (*p* < 0.047, ES: 0.495), with elevated concentrations even 30 min post-exercise. This catabolic response was accompanied by a significant increase in GH under HH (*p* < 0.007, ES: 0.568) and NH (*p* < 0.003, ES: 0.734) as well. Inter-set rest factor had a main effect on C (*p* < 0.006) and GH (*p* < 0.049). No significant main effect was observed for hypoxic environmental condition factor.

The Ca^2+^ level increased significantly in all conditions at every time point after training compared to baseline. A significant main effect was observed for hypoxic environmental condition factor (*p* < 0.038).

The HCO_3_^-^ level decreased after five minutes post-exercise, and the levels remained significantly lowered up to 30 minutes post-exercise in all conditions with a moderate-large effect (>0.90). Concentrations in NH were significantly higher than under HH (*p* < 0.034), and the values after two-minute rest were higher than after one-minute rest (*p* < 0.003).

Pi increased at five minutes post-exercise after training sessions with one-minute rest, both in HH (*p* < 0.001, ES: 0.818) and in NH (*p* < 0.001, ES: 0.974). Then, the values began to decrease progressively and significantly until reaching the baseline at 30 min post-exercise. Both inter-set rest and hypoxic environmental condition factors had a main effect on Pi; values in HH were higher than in NH (*p* < 0.001) and with one-minute than with two-minute rest (*p* < 0.001) as well.

**Figure 1:**
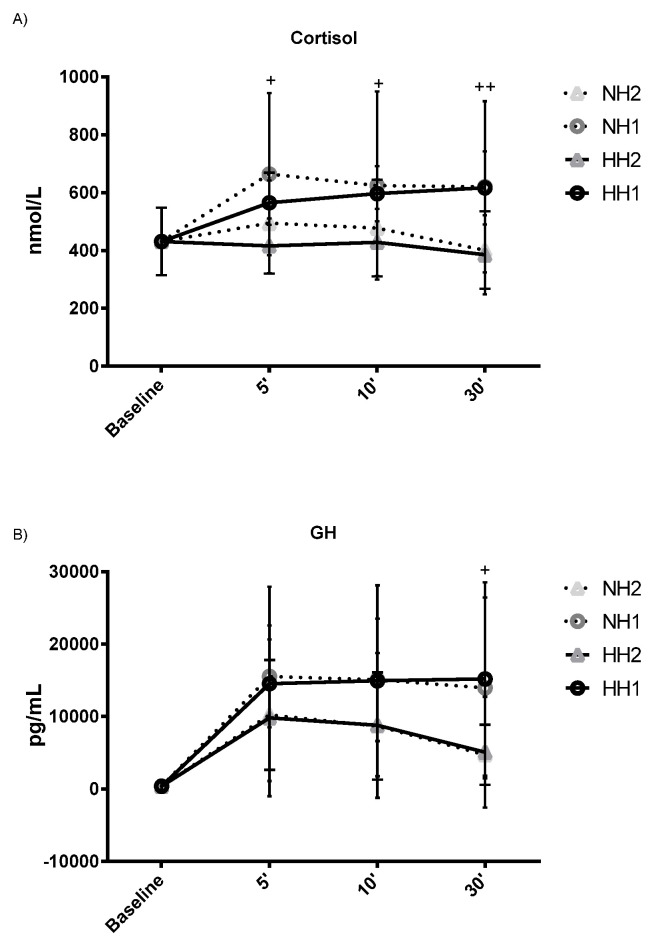
Hormones after resistance training sessions [sub-picture (**A**): cortisol and sub-picture (**B**): GH]. Hypobaric hypoxia (HH) vs. Normobaric hypoxia (NH) with different inter-set rest (1 vs. 2 min). NH2 and NH1: Normobaric Hypoxia with 2-min inter-set rest and 1-min rest. HH2 and HH1: Hypobaric Hypoxia with 2-min inter-set rest and 1-min rest. + *p* < 0.05, ++ *p* < 0.01 comparing 1-min inter-set rest vs. 2 min rest over time. No significant differences were observed between HH vs. NH over time.

**Figure 2:**
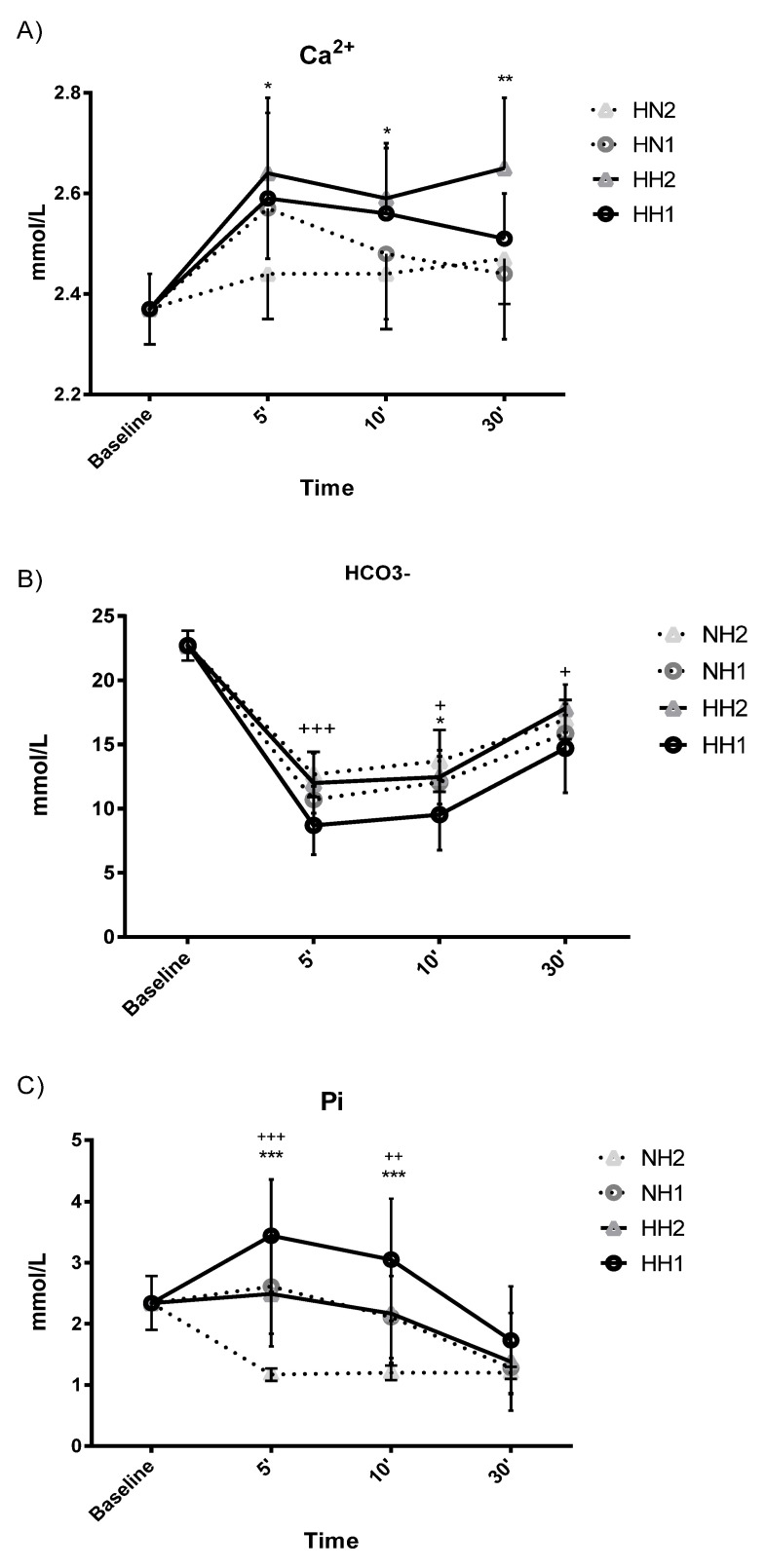
Circulating ions after resistance training sessions [sub-picture (**A**): Ca^2+^, sub-picture (**B**): HCO_3_^−^ and sub-picture (**C**): Pi]. Hypobaric hypoxia (HH) vs. Normobaric hypoxia (NH) with different inter-set rest (1 vs. 2 min). NH2 and NH1: Normobaric Hypoxia with 2-min inter-set rest and 1-min rest. HH2 and HH1: Hypobaric Hypoxia with 2-min inter-set rest and 1-min rest. + *p* < 0.05, ++ *p* < 0.01, +++ *p* < 0.001 comparing 1-min inter-set rest vs. 2 min rest over time. * *p* < 0.05, ** *p* < 0.01, *** *p* < 0.001 comparing HH vs. NH over time.

Figure 1 (hormones) and Figure 2 (circulating ions) specifically show the significant differences that exist over time in both factors (inter-set rest and hypoxic environmental condition). Regarding the inter-set rest factor, the one-minute rest compared to the two-minute rest showed significantly higher values of C (at 5, 10, and 30 min), GH (at 30 min), and of Pi (at 5 and 10 min), and significantly lower of HCO_3_^−^ (at 5, 10, and 30 min). Regarding the hypoxic environmental condition factor, the HH condition showed significantly higher values of Ca^2+^ (at 5, 10, and 30 min) and Pi (at 5 and 10 min), and significantly lower values of HCO_3_^−^ (at 10 min).

## 4. Discussion

HH at terrestrial altitude seems to alter circulating ions related to the muscle contraction more than simulated NH, especially when the inter-set rest intervals are shorter. These findings show that in the design and periodization of high-intensity resistance training in hypoxic environments, it is necessary to pay special attention to the inter-set rest intervals.

High-intensity resistance training causes a significant acute response from serum anabolic and catabolic hormones [24,25]. The results of our study are consistent with these conclusions since an increase in GH levels was observed after training, especially significant when the one-minute inter-set rest was used. The accumulation of lactate and hydrogen ions caused by resistance training stimulates anabolic hormone secretion [17,26]. Likewise, a significant increase in C was observed, although only after training with one-minute inter-set rest. Previous studies have concluded that the use of short inter-set rest caused an increase in metabolic stress [27]. Rahimi et al. [28] concluded that serum C at 30 minutes post-exercise was significantly higher after one-minute inter-set rest than after two-minute rest. In the present research, the use of a hypoxic environment (HH or NH) did not produce any added effect on increasing GH and C levels. Woods et al. [29] stated that performing exercise in HH compared with NN produced a similar pattern of response in C. Similarly, previous studies also did not observe a significant effect of moderate hypoxia on GH and C, either in terrestrial altitude with high-intensity loads [3] or in a simulated altitude with low-intensity loads [12]. The physiological mechanisms behind the hormonal response to hypoxia is unclear and remain speculative. The hormonal changes that occur during acute exercise in hypoxia do not seem to be determined by alterations in the hypothalamic-pituitary axis, but rather by factors related to the workload of the training and the increase in lactate during exercise [30].

Circulating Ca^2+^ levels also increased after resistance training, specifically at five minutes post-exercise, without any differences between training with rest periods of one or two minutes. Resistance training is characterized by repeated skeletal muscle contractions that cause the Ca^2+^ release ions from the sarcoplasmic reticulum into the cytoplasm. This elevation of Ca^2+^ is a consequence of the increase in the concentration of Pi that contributes to its exit from the sarcoplasmic reticulum and reduces the sensitivity of the myofilaments to Ca^2+^ during a first phase of acute fatigue [31,32]. Additionally, it has been shown that intermittent exposure to hypobaric hypoxia can produce a reduction in Ca^2+^ related to the maintenance of calcium homeostasis via activation of protein kinase C isoforms [33]. However, in the present study no differences were observed in Ca^2+^ concentrations between HH and NH. In this sense, factors such as the type, duration, and intensity of exercise play an important role in the disturbance of calcium levels [34], and may be more decisive than the hypoxic environmental factor.

Contrary to what was observed for calcium levels, HCO_3_^−^ levels decreased after training in any hypoxic environment and with both short and long inter-set rest intervals, with a tendency to return to baseline levels after 30 minutes. During intense muscular exercise, there is a significant increase in H^+^ ions. To cope with this acidosis, the cells and fluids of the body have the bicarbonate buffer system, which minimizes the anaerobic production of H^+^ [35]. Therefore, the decrease in HCO_3_^−^ levels indicates a muscle buffering response, especially when the inter-set rest interval is shorter. Additionally, the results also showed that training in HH caused higher decreases in HCO_3_^-^ than training in NH. Compared to NH, HH leads to a greater hypoxemia, blood alkalosis, and a lower SpO_2_ [15]. Likewise, plasma pH appears to be higher in HH than NH [13]. These physiological differences, combined with the exercise-induced metabolic acidosis, could cause changes in acid-base homeostasis and increase the muscle buffering response [36].

Serum Pi increased five minutes after performing both training sessions, in HH and NH, although only after training sessions with one-minute inter-set rest. During exercise, the muscles need energy that is obtained from ATP and cellular phosphocreatine (PCr), whose hydrolysis increases the serum Pi concentration [37]. As in our research, Luhker et al. [36] found elevated Pi levels compared to baseline up to six minutes after exercise in NH conditions (FiO_2_: 0.12). The resynthesis of ATP and the decrease in Pi concentrations appeared to be much faster after training with the longest rest periods (2 min), where at five minutes post-exercise, the levels were even below baseline. These results suggest that the one-minute inter-set rest interval was not sufficient to replenish ATP and PCr, as has been concluded in previous studies [20,38]. Regardless of the inter-set rest interval used, the Pi concentration after training in HH was higher compared to NH training, especially at five minutes post-exercise, but that difference was even maintained until ten minute post-exercise. Several studies have observed a greater decrease in minute ventilation, alveolar ventilation and oxygen saturation [13,15] as well as greater oxidative systemic stress [14,39] with short-term exposure to HH compared to NH. These differences could be the consequence of an increased dead space ventilation, altered fluid permeability or changes in chemo-sensitivity, probably related to the barometric pressure reduction [13]. All these factors could have increased the demands of the resistance training under HH, causing greater fatigue and greater release of Pi in the muscle fiber.

Our study had some limitations. The recovery curve was monitored at specific time points (5, 10, and 30 min post-exercise), and therefore, we have no information about what happened immediately after the training session. We could not control the placebo effect in terrestrial altitude nor in simulated altitude, since these conditions are impossible to dissimulate. However, participants were not previously informed of the expected results after training under hypoxic conditions.

## 5. Conclusions

High-intensity resistance training with one-minute inter-set rest causes greater hormonal stress (C and GH) than with two-minute inter-set rest interval, although HH does not seem to have a significant effect on these hormones. Furthermore, HH at terrestrial altitude seems to alter circulating ions related to the muscle contraction more than simulated NH, especially when the inter-set rest intervals are shorter. These results must be taken into account when planning resistance training in hypoxia, since the inter-set rest interval and the hypoxic environmental condition could be decisive to elicit different metabolic and physiological responses during the training sessions.

## Figures and Tables

**Table 1:** Control variables after training sessions.

Groups	Inter-Set Rest (min)	SpO_2_ (%)	Lactate (mmol/L)	RPE
Hypobaric hypoxia (HH) (*n* = 9)	1	92.44 ± 2.0	19.55 ± 3.5	8.22 ± 1.09
	2	92.77 ± 1.5	15.98 ± 3.89	6.44 ± 1.58 +
Total		92.61 ± 1.7 *	17.7 ± 4.0 *	7.33 ± 1.6
Normobaric hypoxia (NH) (*n* = 7)	1	95.01 ± 1.9	15.30 ± 3.3	7.8 ± 1.21
	2	93.5 ± 2.1	12.47 ± 3.2	6.0 ± 1.73 +
Total		94.28 ± 2.0 *	13.8 ± 3.4 *	6.92 ± 1.7
*p-value*		0.020 *	0.007 *	0.500

* *p* < 0.05. Significant differences between-groups HH vs. NH. + *p* < 0.05. Significant differences within-group (1 min vs. 2 min).

**Table 2:** Ca^2+^, HCO_3_^−^, Pi, and hormones after resistance training session in hypobaric and normobaric hypoxia.

			Hypobaric Hypoxia			Within-Group Factor		Normobaric Hypoxia			Within-Group Factor		Between-Group Factors		
	Rest (min)	Baseline	At 5 min	At 10 min	At 30 min	*p*	ES	At 5 min	At 10 min	At 30 min	*p*	ES	Env (*HH* vs. *NH*)	Rest (*1 m* vs. *2 m*)	Env. x Rest
Ca^2+^ (mmol/L)	1	2.37 ± 0.07	2.59 ± 0.17 *aa*	2.56 ± 0.13 *aa*	2.51 ± 0.09 *aa*	0.004 **	0.599	2.57 ± 0.10 *a*	2.48 ± 0.13	2.44 ± 0.13	0.040 *	0.548	0.038 *	0.197	0.497
	2	2.37 ± 0.07	2.64 ± 0.15 *aa*	2.59 ± 0.11 *aa*	2.65 ± 0.14 *aa*	0.001 **	0.751	2.44 ± 0.09	2.44 ± 0.11	2.47 ± 0.09	*0.333*	0.278			
HCO_3_^−^ (mmol/L)	1	22.70 ± 1.17	8.70 ± 2.28 *aaccdd*	9.53 ± 2.75 *aabbdd*	14.70 ± 3.44 *aabbcc*	0.001 **	0.971	10.70 ± 1.04 *aacdd*	12.05 ± 2.02 *aabdd*	15.86 ± 1.42 *aabbcc*	0.001 **	0.987	0.034 *	0.003 **	0.733
	2	22.70 ± 1.17	11.99 ± 2.33 *aacdd*	12.46 ± 2.10 *aabdd*	17.80 ± 1.86 *aabbcc*	0.001 **	0.965	12.67 ± 1.76 *aaccdd*	13.72 ± 2.40 *aabbdd*	16.94 ± 1.52 *aabbcc*	0.001 **	0.983			
Pi (mmol/L)	1	2.34 ± 0.44	3.44 ± 0.92 *accdd*	3.05 ± 1.00 *bbdd*	1.73 ± 0.88 *bbcc*	0.001 **	0.818	2.61 ± 0.77 *acdd*	2.11 ± 0.67 *bdd*	1.28 ± 0.41 *aabbcc*	0.001 **	0.974	0.001 **	0.001 **	0.211
	2	2.34 ± 0.44	2.49 ± 0.86 *ccdd*	2.17 ± 0.81 *bbdd*	1.38 ± 0.80 *abbcc*	0.001 **	0.688	1.17 ± 0.10 *a*	1.20 ± 0.12 *aa*	1.20 ± 0.10 *aa*	0.001 **	0.719			
C (nmol/L)	1	431 ± 117	565 ± 76 *a*	596 ± 95 *a*	616 ± 126 *a*	0.015*	0.428	664 ± 280 *a*	624 ± 325	620 ± 295	0.047 *	0.495	0.083	0.006 **	0.899
	2	431 ± 117	416 ± 95	428 ± 116 *d*	385 ± 137 *c*	*0.056*	0.407	494 ± 174 *d*	477 ± 167 *d*	401 ± 134 *bc*	*0.370*	0.259			
GH (pg/mL)	1	398 ± 597	14542 ± 13420 *a*	14950 ± 13174 *aa*	15198 ± 13360 *aa*	0.007 **	0.568	15559 ± 7026 *aa*	15091 ± 8464 *aa*	13971 ± 12473 *a*	0.003 **	0.734	0.441	0.049 *	0.997
	2	398 ± 597	9833 ± 10883 *a*	8798 ± 9908	5105 ± 7656	0.028 *	0.466	10248 ± 7572 *ad*	8716 ± 7404	4733 ± 4141 *b*	0.012 *	0.650			

Between-group factors: Hypoxic environmental condition (Env), Inter-set rest (Rest) and Interaction effect: (Env x Rest), * *p* < 0.05 and ** *p* < 0.01. Within-group factor: a: Significant differences compared to baseline, b: Significant differences compared to 5 m; c: Significant differences compared to 10 m; d: Significant differences compared to 30 m. With double symbol of significance: *p* < 0.01; With a single symbol of significance: *p* < 0.05.
